# Diversity and phylogeography of begomovirus-associated beta satellites of okra in India

**DOI:** 10.1186/1743-422X-8-555

**Published:** 2011-12-21

**Authors:** V Venkataravanappa, CN Lakshminarayana Reddy, P Swaranalatha, Salil Jalali, Rob W Briddon, M Krishna Reddy

**Affiliations:** 1 Indian Institute of Horticultural Research, Hessaraghatta Lake PO, Bangalore, India; 2 Indian Vegetable Research Institute, Varanasi 221305, Uttar Pradesh, India; 3 Department of Plant Pathology, College of Sericulture, University of Agricultural Sciences, Chintamani, Karnataka, India; 4 Agricultural Biotechnology Division, National Institute for Biotechnology and Genetic Engineering, P.O. Box 577, Jhang Road, Faisalabad, Pakistan; 5 Division of Plant Pathology, Plant Virology Laboratory, Indian Institute of Horticultural Research, Hessaraghatta Lake PO, Bangalore 560 089, India

**Keywords:** Geminivirus, Begomovirus, Betasatellites, Diversity, Okra, Recombination

## Abstract

**Background:**

Okra (*Abelmoschus esculentus*; family *Malvaceae*) is grown in temperate as well as subtropical regions of the world, both for human consumption as a vegetable and for industrial uses. Okra yields are affected by the diseases caused by phyopathogenic viruses. India is the largest producer of okra and in this region a major biotic constraint to production are viruses of the genus *Begomovirus*. Begomoviruses affecting okra across the Old World are associated with specific, symptom modulating satellites (beta satellites). We describe a comprehensive analysis of the diversity of beta satellites associated with okra in India.

**Results:**

The full-length sequences of 36 beta satellites, isolated from okra exhibiting typical begomovirus symptoms (leaf curl and yellow vein), were determined. The sequences segregated in to four groups. Two groups correspond to the beta satellites Okra leaf curl beta satellite (OLCuB) and Bhendi yellow vein beta satellite (BYVB) that have previously been identified in okra from the sub-continent. One sequence was distinct from all other, previously isolated beta satellites and represents a new species for which we propose the name Bhendi yellow vein India beta satellite (BYVIB). This new beta satellite was nevertheless closely related to BYVB and OLCuB. Most surprising was the identification of Croton yellow vein mosaic beta satellite (CroYVMB) in okra; a beta satellite not previously identified in a malvaceous plant species. The okra beta satellites were shown to have distinct geographic host ranges with BYVB occurring across India whereas OLCuB was only identified in northwestern India. Okra infections with CroYVMB were only identified across the northern and eastern central regions of India. A more detailed analysis of the sequences showed that OLCuB, BYVB and BYVIB share highest identity with respect βC1 gene. βC1 is the only gene encoded by beta satellites, the product of which is the major pathogenicity determinant of begomovirus-beta satellite complexes and is involved in overcoming host defenses based on RNAi.

**Conclusion:**

The diversity of beta satellites in okra across the sub-continent is higher than previously realized and is higher than for any other malvaceous plant species so far analyzed. The beta satellites identified in okra show geographic segregation, which has implications for the development and introduction of resistant okra varieties. However, the finding that the βC1 gene of the major okra beta satellites (OLCuB, BYVB and BYVIB) share high sequence identity and provides a possible avenue to achieve a broad spectrum resistance.

## Background

Geminiviruses are small plant-infecting, arthropod-borne viruses with single-stranded (ss)DNA genomes that are encapsidated in twinned (geminate) quasi-isometric particles. These viruses are found in tropical to warm temperate geographical zones and infect a wide range of plants including crops, ornamental plants and weeds [1]. The family *Geminiviridae *is divided into four genera (*Mastrevirus*, *Curtovirus*, *Topocuvirus *and *Begomovirus*), based on genome structure, type of insect vector and host range. Virus species belonging to the largest genus, *Begomovirus*, are transmitted exclusively by the whitefly *Bemisia tabaci *Genn. and cause economically significant losses of many cultivated dicotyledonous plants.

Begomoviruses native to the New World have genomes that consist of two ssDNA components, known as DNA A and DNA B, each 2.6-2.8 kb in size. The DNA A component encodes all virus factors required for control of gene expression, genome replication and insect transmission between hosts. The DNA B encodes two protein involved in intra- and intercellular movement in host plant tissues [2]. Although a small number of bipartite begomoviruses occur in the Old World, the majority are monopartite, having genomes consisting of only a homolog of the DNA A components of the bipartite viruses. Recently it has become evident that, although there are a few truly monopartite begomoviruses (such as *Tomato yellow leaf curl virus *[3], which has become globally widespread [4]), the majority are monopartite and associate with additional ssDNA molecules [5].

The beta satellites (previously known as DNA β) are large group of highly diverse ssDNA satellites that are approximately half (~1350 nt) the size of their helper begomoviruses and associate with monopartite begomoviruses [5,6]. Despite their lack of sequence conservation, the beta satellites have a highly conserved structure consisting of a single gene (known as βC1), a region of sequence rich in adenine (A-rich) and a sequence of approx. 150 nt highly conserved between all beta satellites (known as the satellite conserved region [SCR]) [7]. Betasatellites require their helper begomoviruses for replication and movement in host plants, as well as for transmission between plants. The relationship between particular begomoviruses and their beta satellites ranges from entirely dependent (the virus has an absolute requirement for the beta satellite to systemically infect a plant host) to facultative (where, in the field, some isolates of a virus species associate with beta satellites and others do not) [8]. All functions thus far attributed to beta satellites are mediated by the product of the βC1 gene. The βC1 protein is a pathogenicity (symptom) determinant [9,10], may mediate virus movement in plants [11], binds DNA in a sequence non-specific manner [12], is a suppressor of RNA silencing (a host defense mechanism targeted against foreign nucleic acids and triggered by double stranded RNA) [12,13], forms homo-multimeric complexes *in planta *[14], interferes with host gene expression [15] and has been shown to interact with a variety of host factors [16,17].

Many begomovirus-beta satellite complexes associate with an additional small ssDNA molecule. Collectively known as alphasatellites (previously called DNA 1) these molecules are satellite-like [5,18]. In common with beta satellites, they are approx. half the size of the genomes of their helper begomoviruses (~1380 nt) and require the helper virus for movement in and transmission between hosts. However, by virtue of encoding a rolling-circle replication-initiator protein (Rep), alphasatellites are capable of autonomous replication in host cells. The benefits to the begomovirus-beta satellite complex of the presence of an alphasatellite remain unclear. In some cases the presence of an alphasatellite has been shown to reduce viral DNA levels leading to the suggestion that alphasatellites may down-regulate virus/satellite replication in hosts, thus ensuring the survival of the plant and consequently increasing the likelihood of onward transmission of the complex [19,20]. However, recently the Rep proteins encoded by some alphasatellites have been shown to have suppressor of RNA silencing activity, suggesting that alphasatellites are involved in overcoming host defenses [21].

We have determined the diversity and phylogeographic distribution of beta satellites associated with okra in India. The results show two species of beta satellite to predominate in okra. An additional beta satellite species, not previously identified in okra, as well as a species not previously characterized were identified. The significance of these results are discussed.

## Results

### Betasatellites are associated with leaf curl and yellow vein disease symptoms of okra in India

**Figure 1:**
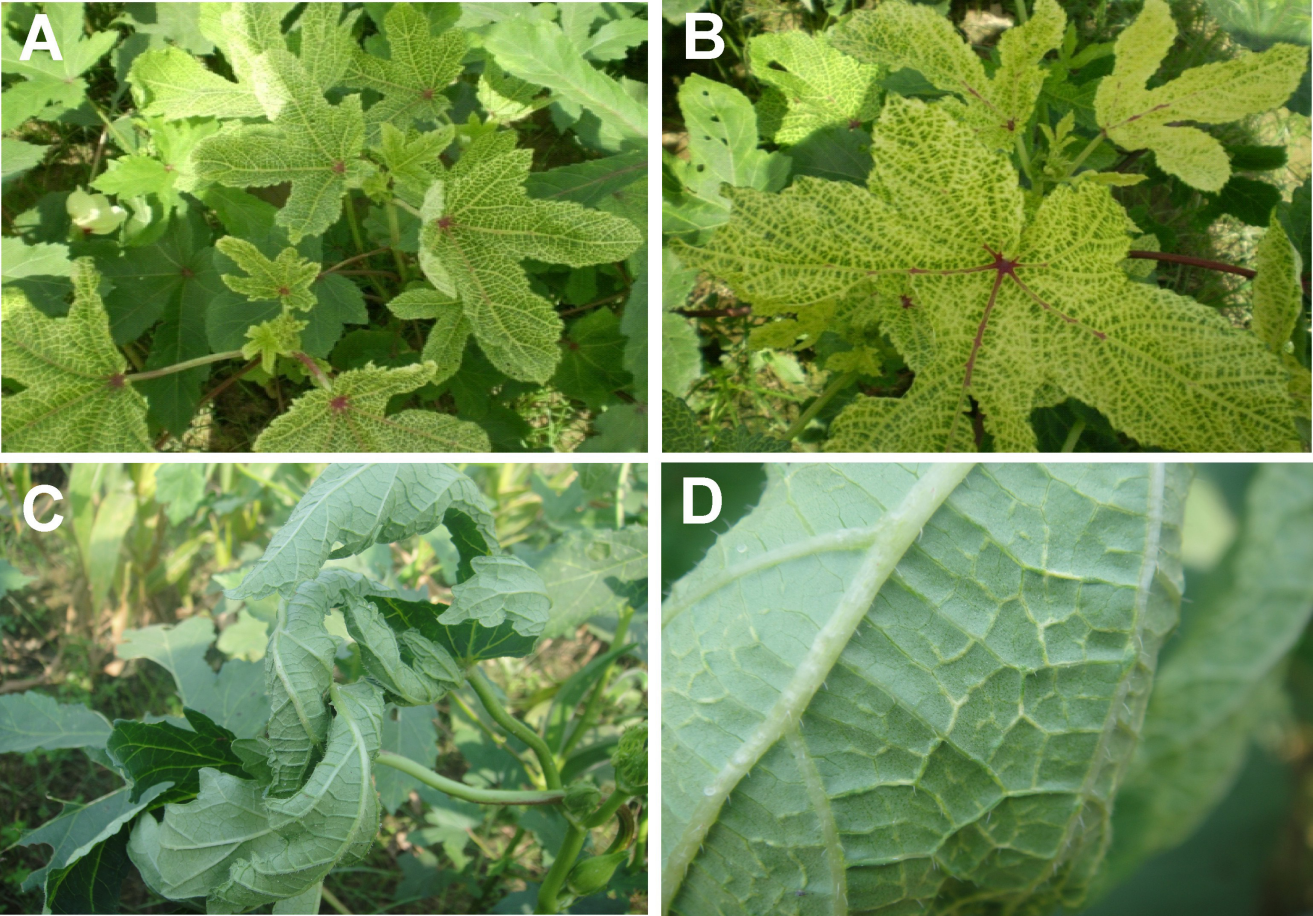
**Symptoms exhibited by okra plants from which betasatellites were obtained**. Yellow vein (panels **a **and **b**) and leaf curl enation (panels **c **and **d**) exhibited by okra plants. Note for the leaf curl enation phenotype the swollen veins (panel **c**) and small enations (tumors) on the secondary veins (panel **d**). Although okra plants with the yellow vein phenotype sometimes showed some leaf curling, this was not associated with the production of enations.

**Figure 2:**
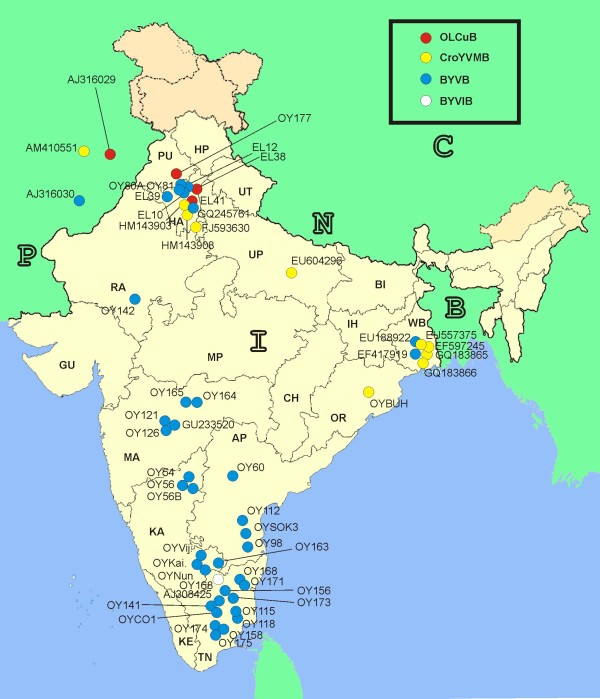
**Geographic origins of okra samples**. Map of southern Asia showing the geographic origins of the okra samples with begomovirus-like symptoms from which betasatellites were cloned. Bhendi yellow vein betasatellite (BYVB) isolates are blue, Okra leaf curl betasatellite (OLCuB) isolates in red and Croton yellow vein mosaic betasatellite (CYVMV) isolates in yellow. The isolates for which betasatellites were determined in this study are indicated by their isolate codes (Table 1) whereas previously determined isolates are indicated by their database accession numbers. The major states India are indicated as Andhra Pradesh (AP), Bihar (BI), Chattisgarh (CH), Gujarat (GU), Haryana (HA), Iharkhand (IR), Karnataka, (KA), Kerala (KE), Madhya Pradesh (MP), Maharashtra (MA), Orissa (OR), Uttaranchal (UT), Uttar Pradesh (UP), Rajasthan (RA), Tamil Nadu (TN) and West Bengal (WB). Countries shown are Bangladesh (B), China (C), India (I), Nepal (N) and Pakistan (P)

A total of 36 leaf samples from okra plants showing yellow vein and/or leaf curl symptoms typical of begomoviruses (Figure 1) were collected from widely separated locations across India between 2005 and 2007 (Table 1, Figure 2). Total nucleic acids were extracted from all symptomatic leaf samples, as well as from non-symptomatic okra samples (two samples from each location). PCR-mediated amplification from nucleic acid extracts of all 36 symptomatic samples with primer pair beta01/beta02 yielded an approx. 1.3 kb product from all samples. In contrast, amplifications from non-symptomatic plants were uniformly negative (results not shown). This result showed a beta satellite to be consistently associated with yellow vein and leaf curl symptoms of okra.

**Table 1:** Origins of okra samples and features of the cloned betasatellites obtained

Isolate	Origin (town, state)	Betasatellite species	**Symptoms [cultivar]**^**▲**^	Database accession number	Size (nt)	Features (coordinates)			βC1 coding capacity	
						
						βC1 gene	SCR	A-rich	No. of amino	Predicted molecular mass (kDa)
OYBHU	Bhubaneswar, Orissa	CroYVMB	Yellow vein mosaic, sever upward leaf curling	GU111995	1344	576-220	142	196	118	12.98

EL38	Sonipat, Haryana	OLCuB	Enation leaf curl,	GU111963	1352	553-197	138	169	118	12.98

EL41	Munthal, Haryana	OLCuB	Enation leaf curl,	GU111965	1360	549-193	138	169	118	12.98

OY177	Chandigarh, Punjab	OLCuB	Yellow vein mosaic, sever upward curling and leaf distortion susceptible line	GU111981	1360	549-193	137	171	118	12.98

OY175	Pandarahalli, Tamil Nadu	BYVB	Enation leaf curl [US Agriseed]	GU111974	1344	669-199	134	214	156	15.95

OY163	Srinivaspur, Karnataka	BYVB	Complete yellowing, petiole bending, vein thickening	GU111992	1372	603-181	136	168	140	16.52

OY164	Aurangabad, Maharashtra	BYVB	Yellow vein mosaic,	GU111988	1385	602-180	138	167	140	15.40

OY126	Jalgov, Maharashtra	BYVB	Leaf crinkling and yellow vein mosaic,	GU111985	1354	536-180	138	170	118	12.98

OY81	Karnal, Haryana	BYVB	Leaf twisting and vein twisting [US 7109]	GU111980	1357	536-180	137	170	118	12.98

EL12	Sonipat, Haryana	BYVB	Enation vein twisting downward curling,	GU111962	1403	689-180	138	170	169	17.27

EL39	Mundhal, Haryana	BYVB	Mild enation	GU111964	1314	718-182	137	164	178	15.40

OY80A	Karnal, Haryana	BYVB	Yellow vein mosaic [Biogauri]	GU111979	1372	767-192	138	176	191	15.40

OY112	Guntur, Andhra Pradesh	BYVB	Intense yellow vein mosaic	GU111969	1356	602-180	137	162	140	15.40

OY54	Raichur, Karnataka	BYVB	Yellow vein mosaic and enation leaf curl	GU111966	1364	683-180	137	214	167	12.98

OY56	Raichur, Karnataka	BYVB	Vein netting and twisting of veins	GU111967	1365	536-180	138	168	118	12.98

OY56B	Raichur, Karnataka	BYVB	Vein netting and twisting of veins	GU111968	1370	535-179	138	214	118	12.98

OY118	Tirchy, Tamil Nadu	BYVB	Yellow vein mosaic	GU111970	1352	536-180^@^	137	168	118^@^	15.95

OY158	Thadagan, Tamil Nadu	BYVB	Yellow vein mosaic and vein thickening,	GU111971	1352	536-180	138	168	118	15.95

OY174	Pandarahalli, Tamil Nadu	BYVB	Yellow vein netting [MH10]	GU111972	1352	536-180	134	168	118	15.95

OYVijapura	Vijaypur (Bijapur), Karnataka	BYVB	Yellow vein mosaic	GU111973	1366	638-180	149	171	152	16.72

OYCO1	Coimbator, Tamil Nadu	BYVB	Yellow vein mosaic [Arun]	GU111975	1373	536-180	134	214	118	12.98

OYKaivara	Kaivara, Karnataka	BYVB	Yellow vein mosaic, leaf distortion	GU111976	1350	735-181	139	168	184	16.61

OY165	Aurangabad, Maharashtra	BYVB	Yellow vein mosaic	GU111977	1354	534-178	138	170	118	12.98

OY60	Guntur, Andhra Pradesh	BYVB	Yellow vein mosaic	GU111982	1376	560-204	138	214	118	12.98

OY115	Tirchy, Tamil Nadu	BYVB	Yellow vein mosaic [MH10]	GU111983	1359	660-187	140	166	157	16.61

OY121	Jalgov, Maharashtra	BYVB	Yellow vein mosaic	GU111984	1358	652-179	138	173	157	16.61

OY141	Coimbator, Tamil Nadu	BYVB	Yellow vein mosaic	GU111986	1381	602-180	134	214	140	12.98

OY156	Thadagan, Tamil Nadu	BYVB	Petiole bending and yellow vein mosaic	GU111987	1357	603-181	134	171	140	15.40

OY171	Dharmapuri, Tamil Nadu	BYVB,	Yellow vein mosaic [MH 10]	GU111989	1351	536-180	138	168	118	14.19

OYNun	Bangalore, Karnataka	BYVB	Yellow vein mosaic, petiole bending	GU111991	1355	740-180	136	168	186	16.72

OYSOK3	Guntur, Andhra Pradesh	BYVB	Yellow vein mosaic and yellow specks	GU111993	1358	536-180	138	172	118	12.98

OY173	Pandarahalli, Tamil Nadu	BYVB	Yellow vein netting	GU111994	1351	536-180	138	167	118	15.95

EL10	Sonipat, Haryana	BYVB	Enation and downward leaf curling	GU111961	1354	602-180	137	168	140	15.40

OY98	Tirupathi, Andhra Pradesh	BYVB	Yellow vein mosaic, petiole bending	GU111978	1352	536-180	138	168	118	12.98

OY142	Udaipur, Rajasthan	BYVB	Yellow vein mosaic [Parbrani Kranthi]	EU081883	1358	536-180	192	182	118	12.98

OY168	Dharmapuri, Tamil Nadu	BYVIB*	Upward leaf curling, vein twisting	GU111990	1383	662-204	134	181	152	16.72

* Isolate of a previously undescribed betasatellite species

^@ ^The βC1 gene is disrupted due to a stop codon resulting from G to A transition at position 476. The corrected information is included in the table

**^▲ ^**Only for a few isolates could the okra cultivar be determined

The approx. 1.3 kb amplification products from all 36 samples were cloned. For each amplification at least one clone containing a presumed full-length (~1.3 kb) beta satellite insert was selected and sequenced in its entirety. The complete nucleotide sequences of 36 beta satellite clones obtained were submitted to the sequence databases under the accession numbers listed in Table 1. The sequences determined are between 1344 and 1403 nt in length, which is typical of beta satellites [6,7]. Further, in order to rule out mixed infections to some extent, in few isolates (including CroYVMB) multiple clones were sequenced. We failed to get the additional beta satellites in these samples.

The 36 cloned beta satellites have an organization similar to previously characterized beta satellites. They contain a highly conserved sequence of 136-142 nts known as the satellite conserved region. This encompasses, at its 3' end, a predicted hairpin structure containing the sequence TAATATTAC (known as the nonanucleotide sequence) within the loop. For geminiviruses this marks the origin of virion-strand, rolling-circle DNA replication which is nicked by virus-encoded Rep to initiate replication [22]. All 36 sequences contain an A-rich sequence of between 164 and 214 nts, with ~60% adenine. Betasatellites encode a single gene (known as βC1), in the complementary-sense strand, which is conserved in position. The βC1 genes in the 36 sequences have a coding capacity of between 118 and 157 amino acids.

### Four beta satellite species are associated with okra in India

Comparisons of the 36 sequences showed them to fall into four groups. The sequences of isolates OY168 and OYBHU are distinct. They share only 39.4% nucleotide sequence identity and show only 61.8-80.2% and 39.6-43.5% identity, respectively, to the remaining 34 sequences. Group 3 consists of three isolates (OY177, EL41 and EL38) which share between 93.9 and 100% identity but only 41.1-68.3% to isolates OY168 and OYBHU, and between 71.2 and 80.6% nucleotide sequence identity to the remaining isolates. The fourth group consists of 31 isolates which have between 76.3 and 99.1% identity. Based on the species demarcation threshold (78%) proposed for beta satellites [6], this indicates that the beta satellites identified in okra in this study represent four distinct species.

A comparison of the sequences of group 4 isolates to betasatellite sequences available in the databases showed them to have the highest levels of nucleotide sequence identity (82-96.4%) to isolates of Bhendi yellow vein betasatellite (BYVB; of which there are six isolates available in the databases) and lower levels of identity (69.7-77.9%) to Okra leaf curl betasatellite (OLCuB; of which there is only a single isolate in the databases; OLCuB-[PK:Goj:97], acc. no. AJ316029 [7]), the two betasatellite species previously shown to be associated with begomoviruses infecting okra in southern Asia [6]. To all other betasatellites the sequence identity of the group 4 isolates was less than 56.3%. This shows the group 4 sequences to represent isolates of the betasatellite species BYVB. Comparisons involving group 3 sequences showed moderately high levels of identity (87.6-88.7%) to OLCuB and lower levels of sequence identity (77.8-80.7%) to BYVB. To all other betasatellites the sequence identity was less than 53.3%, indicating that group 3 sequences are isolates of OLCuB.

Isolate OYBHU showed the highest levels of nucleotide sequence identity (between 84.1 and 92.1%) to isolates of Croton yellow vein mosaic betasatellite (CroYVMB; nine sequences available in the databases) but less than 58.5% to all other betasatellite sequences available in the databases, identifying this as an isolate of the species CroYVMB. This is a surprise since, unlike BYVB and OLCuB, CroYVMB has not previously been shown to infect okra, or any other species in the *Malvaceae*; rather it has been isolated from the ubiquitous weed *Croton bonplandianus *(family *Euphorbiaceae*) and some other weeds [23].

In contrast to all the other betasatellites characterized here, the sequence of isolate OY168 showed only low levels of nucleotide sequence identity to betasatellites available in the databases, with the highest (78%) to BYVB-[IN:Mut:00](AJ308425). Based on the proposed species demarcation threshold of 78% identity for betasatellites [6] this indicates that OY168 represents a new species, for which we propose the name Bhendi yellow vein India betasatellite (BYVIB).

**Figure 3:**
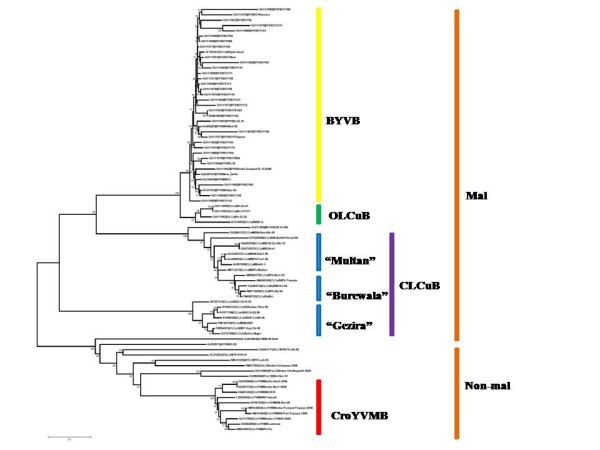
**Phylogeny of Asian betasatellites associated with okra**. Phylogenetic dendrogram based on an alignment of the complete nucleotide sequences of the betasatellites isolated from okra here with all sequences of Bhendi yellow vein betasatellite (BYVB), Okra leaf curl betasatellite (OLCuB) and Croton yellow vein mosaic betasatellite (CroYVMB) available in the databases and selected other betasatellite sequences. The additional betasatellites are Ageratum yellow vein betasatellite (AYVB), Cotton leaf curl Multan betasatellite (CLCuMB; of which there are two variants, the "Multan" and "Burewala" variants [24]), Cotton leaf curl Gezira betasatellite (CLCuGB), Malvastrum yellow vein betasatellite (MaYVB), Tobacco curly shoot betasatellite (TbCSV), Tobacco leaf curl betasatellite (TbLCV) and Tomato yellow leaf curl Thailand betasatellite (TYLCTHB). The virus isolate descriptors for additional sequences are as described in Briddon et al. [6] and the nucleotide sequence database accession numbers of all sequences are given, Numbers at nodes are percentage bootstrap confidence scores (1000 replicates). Malvaceous (Mal) and non-malvaceous (Non-mal) betasatellites, as defined by Briddon et al. [7], are indicated in the right-hand side.

A phylogenetic dendrogram, based upon alignments of the 36 sequences determined here with selected full-length betasatellite sequences available in the databases (including all betasatellites previously identified in okra from southern Asia), supports the aforementioned groupings (Figure 3). Group four isolates segregate with BYVB isolates, group 3 isolates segregate with OLCuB and isolate OYBHU segregates with previously identified CroYVMB sequences. Even though, the betasatellite sequence representing a new species (OY168) based on the nucleotide identity data, it segregates with the BYVBs. The phylogenetic tree here confirms the earlier inclusion of BYVB and OLCuB in the malvaceous betasatellite group and shows that the newly identified species similarly falls in this group. In contrast, CroYVMB is a non-malvaceous betasatellite and would thus not be expected to infect the malvaceous species okra. The phylogenetic analysis (Figure 3) additionally contained sequences of CLCuGB and CLCuMB (of which there are two recognized strains, the "Multan" and "Burewala" strains [24]); the only other betasatellites adapted to a species in the family *Malvaceae *(cotton) for which a significant number of sequences are available. What stands-out here is that the BYVB isolates show overall much higher branch lengths than the CLCuGB and CLCuMB isolates, suggesting that the diversity of BYVB is higher than that of CLCuGB and CLCuMB.

### Analysis of the βC1 sequences of betasatellites associated with okra

**Figure 4:**
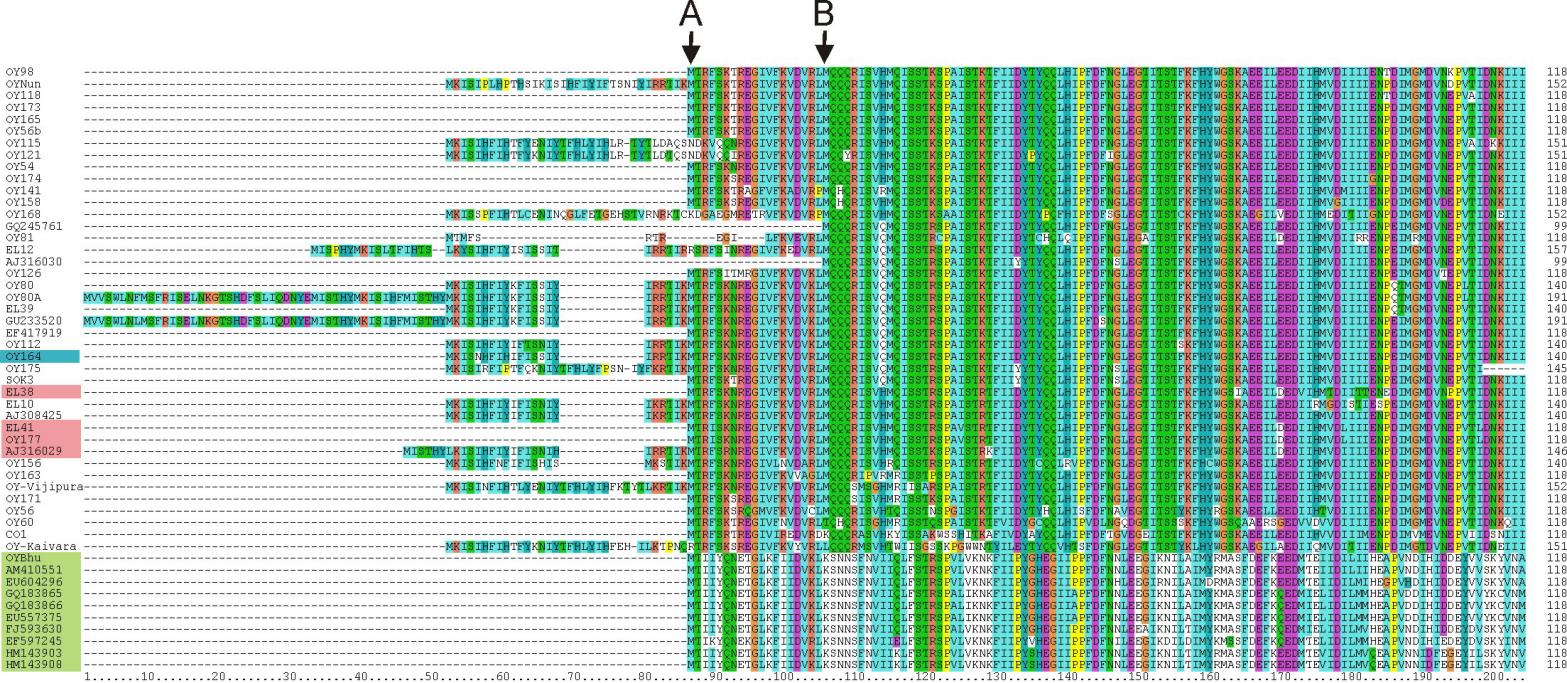
**OLCuB, BYVB and BYVIB encode the same βC1 variant**. Alignment of the predicted amino acid sequences of the βC1 product of the betasatellites identified in okra from India and all Okra leaf curl betasatellite (OLCuB), Bhendi yellow vein betasatellite (BYVB) and Croton yellow vein mosaic betasatellite (CroYVMV) sequences available in the databases. On the left CroYVMV isolates are indicated in green, OLCuB in pink, BYVIB in blue and BYVB isolates are not highlighted. The arrows (marked as A and B) highlight possible translation start codons discussed in the text. The numbers on the right indicate the total numbers of amino acids predicted to be encoded by each βC1 gene.

An alignment of the predicted amino acid sequences of the βC1 genes of the betasatellites characterized here and all BYVB, OLCuB and CroYVMB sequences available in the databases is shown in Figure 4. This shows the majority of the betasatellites under consideration to encode βC1 genes with a coding capacity predicted at 118 amino acids (for the newly characterized betasatellites see Table 1). A small number of the BYVB and OLCuB isolates have extended N-terminal leaders, meaning that they could encode βC1 proteins of a larger size. However, the majority of the betasatellites with extended N-terminal sequences nevertheless encompass the methionine start codon (indicated as A in Figure 4) which would allow for the translation of a βC1 protein of 118 amino acids. Only for two betasatellites, Ageratum yellow vein betasatellite (AYVB) and Cotton leaf curl Multan betasatellite (CLCuMB), have the transcripts spanning the βC1 gene been mapped [9,25]. These transcripts initiate immediately upstream of the methionine equivalent to that marked as A in Figure 4 and would encode a predicted 118 amino acid product. Three isolates of BYVB have βC1 genes with sizes predicted at less than 118 amino acids; BYVB-[PK:Bah:97](AJ316030) and GQ245761 lack the methionine marked A and instead appear possibly to initiate at a highly conserved downstream methionine (marked B in Figure 4) whereas OY175 has a truncation in the C-terminal end of the βC1 gene due to frame shift resulting from the insertion of an A at position 201.

The alignment (Figure 4) shows the sequences of the βC1 proteins of CroYVMB to be distinct from those of both BYVB and OLCuB. However, the βC1 proteins of BYVB and OLCuB appear very similar and also very similar to that of the newly identified BYVIB (OY168). Table 2 shows the variation in βC1 amino acid sequences within and between the betasatellite species. This shows the variation in BYVIB and OLCuB βC1 sequences to fall within the range of variation for BYVB suggesting that these species share highest sequence identity with respect to βC1 gene, even though variation is observed in other part of the genome.

**Table 2:** Range of percent amino acid sequence identities for the βC1 protein excluding the leader sequence

	BYVB (36)*	BYVIB (1)*	OLCuB (4)*	CroYVMB (10)*	CLCuMB (44)*
CLCuMB (44)*	34.2-51.7	45.3-50.8	44.1-51.7	24.8-33.1	80.3-100
CroYVMB (10)*	20.3-31.4	20.3-22.0	27.1-29.7	80.5-100	
OLCuB (4)*	72.0-96.6	75.4-80.5	90.7-100		
BYVIB (1)*	61.9-83.1	-			
BYVB (36)*	57.6-100^@^				

*The figures in brackets indicate the numbers of isolates compared and includes those identified in this study

^@^Three BYVB isolates characterised here (OY60, OYCO1 and OYKaivara) have very unusual βC1 sequences. If these are ignored the range of identities becomes 80.5-100%

### Phylogeographic analysis of okra betasatellites from southern Asia

A map of India with the origins of all betasatellites isolated from okra is shown in Figure 2. This shows BYVB to occur across Indian subcontinent. In contrast, OLCuB appears to be limited to northern India and eastern Pakistan. CroYVMB also has only been identified in the north but appears to have a geographical host range running from Pakistan through the northern states (Uttar Pradesh) to the northeastern coastal states of West Bengal and Orrisa, with OYBUH (from Orrisa) being the most southerly isolate identified thus far.

The phylogenetic tree of betasatellite (Figure 3) shows very low bootstrap scores for the BYVB isolates, even though the values are very high for the distinction (nodes) between BYVB and the other betasatellite sequences. This indicates that there is insufficient information present in the sequences to give a statistically significant placement of the BYVB isolates relative to each other within the tree (in most cases). This likely is due to extensive insertions/deletions of sequence between isolates (results not shown). Conducting the phylogenetic analysis with the "exclude positions with gaps" and "correct for multiple substitutions" options did not significantly alter the topography of the resulting tree, or improve the low bootstrap values, but did significantly reduce branch lengths, particularly for BYVB isolates (results not shown). The analysis thus does not allow us to determine whether there are phylogeographic differences for the distribution of BYVB isolates.

### Analysis for recombination

**Figure 5:**
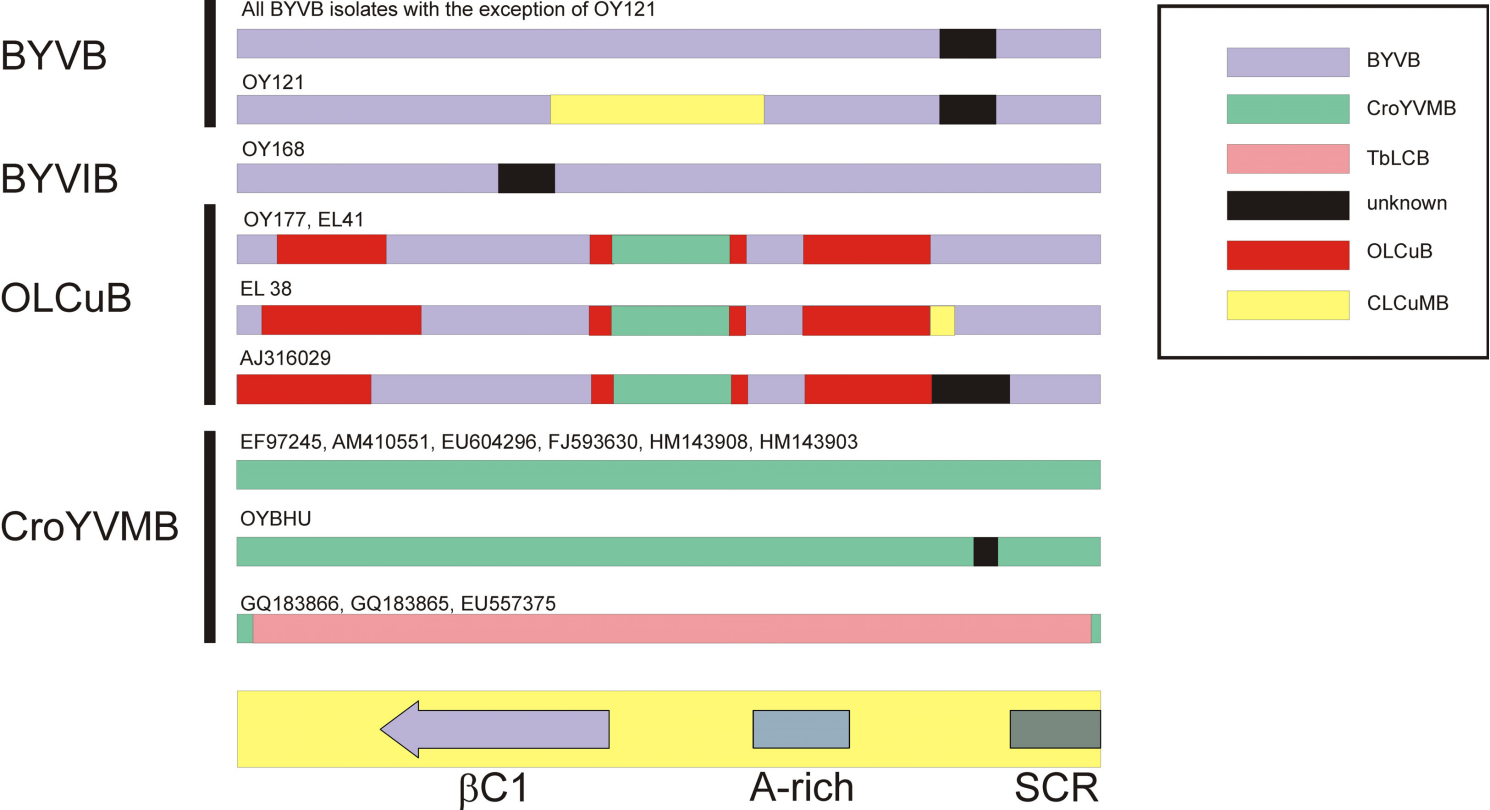
**Analysis of recombination for betasatellites isolated from okra**. The bars represent the sequences of betasatellites with the likely origins of sequences shown in color, as indicated in the box on the right. Only interspecific recombinations, that is recombination between distinct betasatellites (as defined by Briddon et al. [6]), are shown. The betasatellite acronyms given are Bhendi yellow vein betasatellite (BYVB), Croton yellow vein mosaic betasatellite (CroYVMB), Cotton leaf curl Multan betasatellite (CLCuMB), Okra leaf curl betasatellite (OLCuB) and Tobacco leaf curl betasatellite (TbLCB). Sequence of indeterminate origin is indicated as "unknown". The box below at the bottom of the diagram indicates the approximate position of the βC1 gene, the A-rich region and the satellite conserved region (SCR), features that are common to all betasatellites.

The results of a comprehensive analysis, using the Recombination detection program (RDP; [26]) and based upon an alignment of all betasatellite sequences obtained here with selected other betasatellites from the databases, is summarized in Additional file 1: Table S1. The major interspecific recombination events are summarized in Figure 5. The analysis showed the majority of BYVB sequences to show little evidence of recombination. Only for one isolate (OY121) was there evidence for possible recombination with an unrelated betasatellite, CLCuMB. The isolate shown to represent a new species of betasatellite, OY168, showed little evidence of recombination, consisting for the most part of sequence derived from BYVB and some sequence of indeterminate parentage. In contrast, all three OLCuB isolates showed very similar recombination patterns, with BYVB as the major parent, some sequence apparently unique to OLCuB isolates and a common fragment possibly derived from CroYVMB. This is consistent with the common geographic distribution of BYVB, OLCuB and CroYVMB. This suggests that OLCuB is a recombinant derivative of BYVB and CroYVMB.

The CroYVMB isolate (OYBHU) consists for the most part of sequence unique to CroYVMB with some sequence of unknown origin. It is interesting to note that three CroYMV isolates originating from the far east of the country (GQ183865, GQ183866 and EU557375) are distinct from all other CroYVMV isolates in having much of their sequence apparently derived from a Tobacco leaf curl betasatellite (TbLCB). In contrast to the other CroYVMB isolates, which were isolated from *croton*, papaya, radish and *jatropha*, these three isolates came from the legume *Crotalaria juncea*, often used as a green manure and fodder crop. This may suggest the *C. juncea *is selecting for a distinct recombinant CroYVMB.

## Discussion

The study presented here has greatly extended our knowledge of the diversity of betasatellites associated with begomovirus disease of okra across Indian subcontinent. Prior to this study only a single OLCuB and six BYVB isolates had been characterized. Our analysis shows BYVB to be the major betasatellite associated with okra and that this occurs across the whole of southern Asia. OLCuB appears to have a more limited geographic distribution across northern Pakistan and northern India.

The malvaceous plants in the New World are affected by bipartite begomoviruses [27-29] betasatellites not apparently occurring in the New World [7]. In the Old World begomoviruses infecting okra are invariably associated with betasatellites and are, for the few which have been characterized, monopartite--lacking the DNA B component. Across Africa okra and other malveaceous hosts are affected by is affected by leaf curl disease, which is associated with begomoviruses and a single betasatellite, Cotton leaf curl Gezira betasatellite (CLCuGB) [30-32]. Although first identified in cotton [33], CLCuGB is also widespread in hollyhock, *Sida *spp. and tomato [7,34,35]. This situation in Africa, apparently a single betasatellite affecting okra, contrasts markedly with the different betasatellites affecting situation we have shown in India. Okra, as we have shown is affected by distinct betasatellites. There is at this time some debate as to the geographic origins of okra, with the majority favoring a southern Asian origin over a North African origin [36]. The evidence presented here, a greater diversity of betasatellites of okra in India than in Africa suggesting a longer association of these on the sub-continent, might add weight to this argument. This is in agreement with the conclusions of Nawaz-ul-Rehman and Fauquet [37] who showed the center of diversity, and thus likely the center of origin, of begomoviruses and betasatellites to reside in Southeast Asia.

The presence of CroYVMB has been shown in different host plants belonging to non- malvaceous plants such as, *Croton bonplandianus *(EF597245), *Croton *sp. (AM410551), *Crotalaria juncea *(GQ183865, GQ183866, EU557375), radish (FJ593630), *Jatropha gossypifolia *(EU604296) and papaya (HM143903, HM143908), which are supported by different helper begomoviruses [23,38]. However, the identification of CroYVMB in okra is something of a conundrum. CroYVMB is not a "malvaceous betasatellite" and has not previously been identified in any malvaceous plant species. The possible explanation for this may just be phenomenon known as pathogen "reassortment" due to insect transmission by *B. tabaci*, vector of a pathogen from another host plant that is maintained *in trans *by a helper begomovirus.

Betasatellites, on the whole, fall into two distinct groups, those isolated from species of the family *Malvaceae *and those isolated from non-malvaceous species [5,7]. Although the malvaceous betasatellites are frequently identified in non-malvaceous plants, the converse has not been reported so frequently. This has been taken to indicate that the requirements for infection of species in the family *Malvaceae *differ from those for non-malvaceous species.

With three (possibly four) distinct betasatellites associated with disease in okra, this raises the question as to whether they induce distinct symptoms? Based on the data in Table 1, there is no clear correlation between symptom type (leaf curl/enation or yellow vein) and a particular betasatellite species. The leaf curl/enation and yellow vein phenotypes are associated with infections of both OLCuB and BYVB. Of course, since only single clones were characterized from each sample (multiple clones were characterized in only few isolates (data not shown)), it is possible that the major betasatellite (of a mixed infection) was not characterized, leading to deceptive results. However, for CLCuMB it has been shown that βC1 can phenocopy all symptoms of the disease (CLCuD) when introduced into a plant using a *Potato virus X *vector [39], showing that the major symptom determinant, at least for the CLCuD begomovirus-betasatellite, not the virus. It would thus be somewhat surprising to find three distinct betasatellite species that share a high level of sequence identity with respect to βC1 gene could induce distinct symptoms. The possible reasons for this may be attributed to helper viruses, other betasatellites in mixed infections, or okra varieties. Again, only experimental inoculation with defined clones will provide a definitive answer to this question.

The finding that the leaf curl/enation phenotype for infections of okra is restricted to Pakistan and northern Indian is puzzling. The possible reason reasons for this may be again, the helper virus, other betasatellites in mixed infections, okra varieties or that some other factor, such as for example co-infection with another (as yet unidentified) virus or satellite (-like) components determine the symptom differences. Recently two studies have shown that alphasatellites, the third partner in many begomovirus-betasatellite infections, can have a significant effect on symptoms [21,40], although this did not lead to a change in symptom phenotype, merely an amelioration in symptom severity. It is also interesting to note that the geographical occurrence of the leaf curl/enation phenotype in okra overlaps the range of CLCuD and one of the viruses (*Cotton leaf curl Multan virus*), as well as the betasatellite know to cause CLCuD (CLCuMB), has recently been implicated in okra leaf curl disease for the first time reported from China [41].

The identification of a relatively high level of diversity of betasatellites in okra has implications for the development of resistance, by both conventional breeding and non-conventional (transgenic) approaches. Any resistant varieties produced will need to be able to counter begomoviruses supporting all possible betasatellites to have any chance of being durable. The finding that three of the betasatellites identified in okra, OLCuB, BYVB and BYVIB, have a βC1 gene with high sequence identity provides a possible means of achieving a broad spectrum resistance in okra to begomovirus-betasatellite diseases. Targeting the shared βC1 gene (by for example RNAi -mediated down regulation of transcript levels) or the product of the βC1 gene (using, for example, peptide aptamers [42,43]) might yield a resistance active against three of the four betasatellites identified here--"one stone killing three birds".

Our efforts are now centered on analyzing the diversity of begomoviruses that occur in okra in India. Only a single virus, BYVV, has thus far been identified in okra in association with BYVB [44]. The results indicated association of multiple betasatellites which are supported by multiple distinct helper begomoviruses. We have identified at least three distinct begomoviruses associated with okra in India (unpublished).

## Materials and methods

### Collection of okra samples

Live okra plants, exhibiting virus-like symptoms, were collected from across India during 2005 and 2007. The geographical origin, okra cultivar (where known) and symptoms exhibited by the plants are summarized in Table 1. The plants were maintained in an insect-free glasshouse at 28°C with supplementary lighting to yield a 16 h photoperiod.

### DNA extraction, PCR amplification, cloning and sequencing

Total Nucleic acid was extracted from field-collected okra leaf samples using the CTAB method as modified by Lodhi et al. [45]. Full-length betasatellites were amplified by PCR using universal primers (β01/β02; [46]). These primers were designed to allow the amplification of the entire betasatellite and have been shown previously to produce products which are, in many cases, infectious to plants [7,47]. The amplified product was cloned into the pTZ57R vector (Fermentas) according to manufacturer's instruction. Transformation was performed using DH5α *Escherichia coli *cells. Nucleotide sequences of plasmid DNA from clones were determined by automated sequencing at Anshul Biotechnologies DNA Sequencing core laboratory (Hyderabad, India).

### Sequence analysis

Nucleic acid sequences were analyzed using the Basic Local Alignment Search Tool (BLAST, NCBI) to search for similar sequences in the database. Multiple alignments were performed using Clustal X [48], percentage of sequence identities were obtained using bioedit (version 7.0.9) and the neighbor joining phylogenetic tree was generated using MEGA5 with 1000 bootstrap replications [49] and pairwise evolutionary distances were calculated with a maximum composite likelihood nucleotide substitution model. Analysis for recombination used the Discreet recombination events were detected using the RDP [26], GENECONV [50], MAXCHI [51], CHIMAERA [52], SISCAN [53], and 3SEQ [54] methods implemented in the program RDP3 (version 3.44; available from http://darwin.uvigo.es/rdp/rdp.html) [26]. Default RDP3 settings with a 0.05 *P-value *cutoff with standard Bonferroni correction for multiple testing were used throughout.

## Competing interests

The authors declare that they have no competing interests.

## Authors' contributions

VV performed the experiments. VV, CNLR, PS, SL, RWB and MKR were involved in data analysis. MKR provided overall direction and experimental design. VV, CNL, MKR and RWB wrote the manuscript. All authors read and approved the final manuscript.

## Supplementary Material

Additional file 1 **Table S1**. Recombination events detected in the betasatellites identified in this study including approximate breakpoint positions, parental-like sequences, and p-values for various recombination detection tests. Click here for file
