# Supplementary material for: Diversity and phylogeography of begomovirus-associated beta satellites of okra in India
Source: Virol J. 2011 Dec 21;8:555. doi: 10.1186/1743-422X-8-555 (PMC3267694; doi:10.1186/1743-422X-8-555)
Supplement: Additional file 1 — Table S1. Recombination events detected in the betasatellites identified in this study including approximate breakpoint positions, parental-like sequences, and p-values for various recombination detection tests. [file 1743-422X-8-555-S1.DOC]

Supplemantary Table 1. Recombination events detected in the betasatellites identified in this study including approximate breakpoint positions, parental-

like sequences, and p-values for various recombination detection tests.

| Isolate | Break point begin-end | Major Parent | Minor parent | RDP | GENECOV | Max Chi | Chimera | Si Scan | 3Seq |
| --- | --- | --- | --- | --- | --- | --- | --- | --- | --- |
| OYBHU | 1110-1125 | CroYVMB-Lucknow(EU604296) | BYVB-[IN:Bih9:10]( GU732208) | 2.327X10^-13^ | 8.149X10^-8^ | 7.635X10^-4^ | NS | NS | NS |
| OY177 | 1131-40  231-614  644-796  810-888 | OY Vijipura (GU111973)  OYKaivara (GU111976)  OY168 (GU111990)  OYSOK3 (GU111992) | OY80(GU111979)  BYVB-[IN:MUT:00]( AJ308425)  CroYVMB-Lucknow(EU604296)  BYVB-[IN:MUT:00]( AJ308425) | NS  1.292X10^-4^  NS  4.754X10^-7^ | NS  4.024X10^-17^  NS  4.021X10^-4^ | 1.509X10^-2^  2.466X10^-11^  2.846X10^-4^  NS | 4.13X10^-2^  1.540X10^-12^  3.641X10^-3^  NS | 3.719X10^-6^  9.287X10^-18^  NS  4.961X10^-5^ | 7.209X10^-4^  1.233X10^-23^  NS  2.036X10^-3^ |
| OY174 | 657-26  1111-1177 | OY56b (GU111968)  CLCuMB-[IN:DAB1:95]( AJ316037) | OYKaivara (GU111976)  TbLCB-[PK:BAH:99]( AJ316034) | NS  NS | 8.757X10^-9^  NS | 3.5X10^-11^  1.105X10^-2^ | 7.449X10^-12^  3.453X10^-3^ | 6.249X10^-18^  NS | 8.735X10^-27^  1.853X10^-2^ |
| OY175 | 101-549  1108-1179 | OY121 (GU111984)  CLCuMB-[IN:DAB1:95]( AJ316037) | OYSOK3 (GU111992)  TbLCB-[PK:BAH:99]( AJ316034) | NS  NS | NS  NS | 1.096X10-2  1.105X10-2 | 2.874X10-3  3.453X10-3 | NS  NS | 1.18X10-4  1.853X10-2 |
| OY112 | 902-27  1114-1180 | OY60 (GU111982)  CLCuMB-[IN:DAB1:95]( AJ316037) | OYSOK3 (GU111992)  TbLCB-[PK:BAH:99]( AJ316034) | 1.236X10^-3^  NS | 2.615X10^-6^  NS | 1.259X10^-5^  1.105X10^-2^ | 1.769X10^-5^  3.453X10^-3^ | NS  NS | 1.623X10^-11^  1.853X10^-2^ |
| OY164 | 856-52  1149-1215 | OY60 (GU111982)  CLCuMB-[IN:DAB1:95]( AJ316037) | OYSOK3 (GU111992)  TbLCB-[PK:BAH:99]( AJ316034) | 1.236X10^-3^  NS | 2.605X10^-6^  NS | 1.259X10^-5^  1.105X10^-2^ | 1.769X10^-5^  3.453X10^-3^ | 1.623X10^-11^  NS | NS  1.853X10^-2^ |
| OYVijapura | 1109-1180 | CLCuMB-[IN:DAB1:95]( AJ316037) | TbLCB-[PK:BAH:99]( AJ316034) | NS | NS | 1.105X10^-2^ | 3.453X10^-3^ | NS | 1.853X10^-2^ |
| OY156 | 1111-1407 | CLCuMB-[IN:DAB1:95]( AJ316037) | TbLCB-[PK:BAH:99]( AJ316034) | NS | NS | 1.105X10^-2^ | 3.453X10^-3^ | NS | 1.853X10^-2^ |
| OY80 | 419-805  1126-1197 | OY126 (GU111985)  CLCuMB-[IN:DAB1:95]( AJ316037) | BYVB-[IN:Bar:06]( EF417919)  TbLCB-[PK:BAH:99]( AJ316034) | NS  NS | 1.068X10^-8^  NS | 4.691X10^-9^  1.105X10^-2^ | 4.106X10^-9^  3.453X10^-3^ | 2.529X10^-9^  NS | 1.93X10^-18^  1.853X10^-2^ |
| OY81 | 747-1101  797-1253 | OY81 (GU111979)  CLCuMB-[IN:DAB1:95]( AJ316037) | OY163 (GU111993)  TbLCB-[PK:BAH:99]( AJ316034) | NS  NS | 4.965X10^-5^  NS | 1.237X10^-3^  1.105X10^-2^ | 1.003X10^-3^  3.453X10^-3^ | 6.256X10^-6^  NS | 1.654X10^-5^  1.853X10^-2^ |
| OY126 | 735-111  509-717  1107-1178 | BYVB.[IN.Kar.06] OYSOK3 (GU111992)  GU233520  CLCuMB-[IN:DAB1:95]( AJ316037) | OY163 (GU111993)  OY81 (GU111979)  TbLCB-[PK:BAH:99]( AJ316034) | NS  NS  NS | 4.965X10^-5^  NS  NS | 1.237X10^-3^  5.497X10^-3^  1.105X10^-2^ | 1.003X10^-3^  NS  3.453X10^-3^ | 6.256X10^-6^  9.639X10^-5^  NS | 1.654X10^-5^  NS  1.853X10^-2^ |
| OY60 | 26-523  418-1317  1130-1201 | OY54 (GU111966)  OY156 (GU111987)  CLCuMB-[IN:DAB1:95]( AJ316037) | OY168 (GU111990)  OYnun(GU111991)  TbLCB-[PK:BAH:99]( AJ316034) | NS  NS  NS | NS  3.569X10^-2^  NS | NS  3.805X10^-7^  1.105X10^-2^ | NS  1.182X10^-7^  3.453X10^-3^ | 6.753X10^-9^  6.682X10^-29^  NS | 2.735X10-2  7.975X10^-14^  1.853X10^-2^ |

Supplementary Table 1. Continued

| Isolate | Break point begin-end | Major Parent | Minor parent | RDP | GENECOV | Max Chi | Chimera | Si Scan | 3Seq |
| --- | --- | --- | --- | --- | --- | --- | --- | --- | --- |
| OY115 | 256-119  1110-1182 | OY156 (GU111987)  CLCuMB-[IN:DAB1:95]( AJ316037) | OYnun(GU111991)  TbLCB-[PK:BAH:99]( AJ316034) | NS  NS | 3.569X10^-2^  NS | 3.805X10^-7^  1.105X10^-2^ | 1.182X10^-7^  3.453X10^-3^ | 6.682X10^-29^  NS | 7.975X10^-14^  1.853X10^-2^ |
| OY141 | 664-99  111-464  1138-1210 | OY173 (GU111994)  OY115 (GU111983)  CLCuMB-[IN:DAB1:95]( AJ316037) | OYCO1 (GU111975)  OY60 (GU111982)  TbLCB-[PK:BAH:99]( AJ316034) | NS  NS  NS | 6.042X10^-16^  NS  NS | 9.969X10^-15^  NS  1.105X10^-2^ | 1.708X10^-9^  NS  3.453X10^-3^ | 3.07X10^-18^  2.938X10^-5^  NS | 3.891X10^-18^  2.183X10^-2^  1.853X10^-2^ |
| OYCO1 | 779-107  1126-1198 | OY175 (GU111974)  CLCuMB-[IN:DAB1:95]( AJ316037) | OYSOK3 (GU111992)  TbLCB-[PK:BAH:99]( AJ316034) | 2.4X10^-3^  NS | 6.611X10^-6^  NS | 4.611X10^-5^  1.105X10^-2^ | 3.690X10^-10^  NS | 2.953X10^-10^  NS | 1.372X10^-8^  1.853X10^-2^ |
| OY56B | 773-1311  1124-1195 | OY175 (GU111974)  CLCuMB-[IN:DAB1:95]( AJ316037) | OYSOK3 (GU111992)  TbLCB-[PK:BAH:99]( AJ316034) | 2.4X10^-3^  NS | 6.611X10^-6^  NS | 4.611X10^-5^  1.105X10_-2_ | 3.690X10^-10^  3.453X10^-3^ | 2.953X10^-10^  NS | 1.372X10^-8^  1.853X10^-2^ |
| OY168 | 528-615  1155-1245 | OLCuB-[PK:Goj:03]( AJ316029)  OY81 (GU111979) | CLCuMB(AM774309)  AJ308425|BYVB.[IN.MUT:00] | NS  NS | NS  9.038X10^-7^ | 2.27X10^-6^  NS | 5.745X10^-3^  NS | NS  6.493X10^-3^ | NS  NS |
| OYKaivara | 93-592  373-505  1105-1176 | OY175 (GU111974)  OY56 (GU111967)  CLCuMB-[IN:DAB1:95]( AJ316037) | OYSOK3 (GU111992)  OY168 (GU111990)  TbLCB-[PK:BAH:99]( AJ316034) | 2.4X10^-3^  NS  NS | 6.611X10^-6^  NS  NS | 4.611X10^-5^  5.963X10^-5^  1.105X10^-2^ | 3.690X10^-10^  1.084X10^-5^  3.453X10^-3^ | 2.953X10^-10^  NS  NS | 1.372X10^-8^  1.011X10^-6^  1.853X10^-2^ |
| OY121 | 934-655  1112-1183 | OYKaivara (GU111976)  CLCuMB-[IN:DAB1:95]( AJ316037) | OY56b (GU111968)  TbLCB-[PK:BAH:99]( AJ316034) | NS  NS | 8.236X10-9  NS | 1.638X10^-12^  1.105X10^-2^ | 1.267X10^-12^  3.453X10^-3^ | 4.247X10^-19^  NS | 3.661X10^-29^  1.853X10^-2^ |
| EL39 | 910-78  409-756  1068-1139 | OY80 (GU111979)  BOY126 (GU111985)  EL39(GU111964) | OY165(GU111977)  BYVB-[IN:Bar:06]( EF417919)  CLCuMB-[IN:DAB1:95]( AJ316037) | 2.857X10^-2^  NS  NS | NS  1.068X10^-8^  NS | 1.499X10^-5^  4.691X10^-9^  1.105X10^-2^ | 8.264X10^-4^  4.106X10^-9^  3.453X10_-3_ | NS  2.529X10^-12^  NS | 9.484X10^-9^  1.930X10^-18^  1.853X10^-2^ |
| EL41 | 1131-40  231-614  644-769  810-888 | OY Vijipura (GU111973)  OYKaivara (GU111976)  OY168 (GU111990)  OYSOK3 (GU111992) | BOY81 (GU111979)  BYVB-[IN:MUT:00]( AJ308425)  CroYVMB-Lucknow(EU604296)  BYVB-[IN:MUT:00]( AJ308425) | NS  1.292X10^-4^  NS  4.754X10^-7^ | NS  4.024X10^-17^  NS  4.021X10^-4^ | 1.509X10^-2^  2.466X10^-11^  2.846X10^-4^  NS | 4.013X10^-2^  1.54X10^-12^  3.641X10^-3^  NS | 3.719X10^-2^  9.287X10^-18^  NS  4.961X10^-5^ | 7.209X10^-4^  1.233X10^-23^  NS  2.036X10^-3^ |
| EL12 | 476-1093  1157-1228 | OY164(GU111988)  CLCuMB-[IN:DAB1:95]( AJ316037) | OY115 (GU111983)  TbLCB-[PK:BAH:99]( AJ316034) | NS  NS | 5.221X10^-8^  NS | 4.092X10^-10^  1.105X10^-2^ | 5.803X10^-12^  3.453X10^-3^ | 6.772X10^-15^  NS | 1.268X10^-21^  1.853X10^-2^ |
| EL38 | 1125-30  303-606  647-790  804-882 | OY81 (GU111979)  OYKaivara (GU111976)  OY168 (GU111990)  OYSOK3 (GU111992) | OY Vijipura (GU111973)  BYVB-[IN:MUT:00]( AJ308425)  CroYVMB-Lucknow(EU604296)  BYVB-[IN:MUT:00]( AJ308425) | NS  1.292X10^-4^  NS  4.754X10^-7^ | NS  4.024X10^-17^  NS  4.021X10^-4^ | 1.509X10^-2^  2.466X10^-11^  2.846X10^-4^  NS | 4.013X10^-2^  1.54X10^-12^  3.641X10^-3^  NS | 3.719X10^-2^  9.287X10^-18^  NS  4.961X10^-5^ | 7.209X10^-4^  1.233X10^-23^  NS  2.036X10^-3^ |

Supplementary Table 1. Continued

| Isolate | Break point begin-end | Major Parent | Minor parent | RDP | GENECOV | Max Chi | Chimera | Si Scan | 3Seq |
| --- | --- | --- | --- | --- | --- | --- | --- | --- | --- |
| EL10 | 334-605  1108-1179 | OY142(EU081883)  CLCuMB-[IN:DAB1:95]( AJ316037) | BYVB-[IN:MUT:00]( AJ308425)  TbLCB-[PK:BAH:99]( AJ316034) | NS  NS | NS  NS | 2.901X10^-3^  1.105X10^-2^ | NS  3.453X10^-3^ | 6.927X10^-6^  NS | NS  1.853X10^-2^ |
| OY163 | 619-791  1110-1182 | CroYVMB-Lucknow(EU604296)  CLCuMB-[IN:DAB1:95]( AJ316037) | OY168 (GU111990)  TbLCB-[PK:BAH:99]( AJ316034) | NS  NS | NS  NS | 2.846X10^-4^  1.105X10^-2^ | 3.641X10^-3^  3.453X10^-3^ | NS  NS | NS  1.853X10^-2^ |
| OY158 | 107-540  1106-1177 | OYSOK3 (GU111992)  CLCuMB-[IN:DAB1:95]( AJ316037) | OY175 (GU111974)  TbLCB-[PK:BAH:99]( AJ316034) | 2.4X10^-3^  NS | 6.611X10-6  NS | 4.611X10^-5^  1.105X10^-2^ | 3.690X10^-10^  3.453X10^-3^ | 2.953X10^-10^  NS | 1.372X10^-8^  1.853X10^-2^ |
| OY171 | 107-492  1105-1176 | OYSOK3 (GU111992)  CLCuMB-[IN:DAB1:95]( AJ316037) | OY175 (GU111974)  TbLCB-[PK:BAH:99]( AJ316034) | 2.4X10^-3^  NS | 6.611X10^-6^  NS | 4.611X10^-5^  1.105X10^-2^ | 3.690X10^-10^  3.453X10^-3^ | 2.953X10^-10^  NS | 1.372X10^-8^  1.853X10^-2^ |
| OY98 | 890-1293  1106-1177 | OYSOK3 (GU111992)  CLCuMB-[IN:DAB1:95]( AJ316037) | OY175 (GU111974)  TbLCB-[PK:BAH:99]( AJ316034) | 2.4X10^-3^  NS | 6.611X10^-6^  NS | 4.611X10^-5^  1.105X10^-2^ | 3.690X10^-10^  3.453X10^-3^ | 2.953X10^-10^  NS | 1.372X10^-8^  1.853X10^-2^ |
| OY118 | 373-491  1106-1177 | OY175 (GU111974)  CLCuMB-[IN:DAB1:95]( AJ316037) | OYSOK3 (GU111992)  TbLCB-[PK:BAH:99]( AJ316034) | 2.4X10^-3^  NS | 6.611X10^-6^  NS | 4.611X10^-5^  1.105X10^-2^ | 3.690X10-^10^  3.453X10^-3^ | 2.953X10^-10^  NS | 1.372X10^-8^  1.853X10^-2^ |
| OY173 | 619-791  1105-1176 | CroYVMB-Lucknow(EU604296)  CLCuMB-[IN:DAB1:95]( AJ316037) | OY168 (GU111990)  TbLCB-[PK:BAH:99]( AJ316034) | NS  NS | NS  NS | 2.846X10^-4^  1.105X10^-2^ | 3.641X10^-3^  3.453X10^-3^ | NS  NS | NS  1.853X10^-2^ |
| OY165 | 1108-1179 | CLCuMB-[IN:DAB1:95]( AJ316037) | TbLCB-[PK:BAH:99]( AJ316034) | NS | NS | 1.105X10^-2^ | 3.453X10^-3^ | NS | 1.853X10^-2^ |
| OY54 | 767-1282  1118-1190 | OY175 (GU111974)  CLCuMB-[IN:DAB1:95]( AJ316037) | OYSOK3 (GU111992)  TbLCB-[PK:BAH:99]( AJ316034) | 2.4X10^-3^  NS | 6.611X10^-6^  NS | 4.611X10^-5^  1.105X10^-2^ | 3.690X10^-10^  3.453X10^-3^ | 2.953X10^-10^  NS | 1.372X10^-8^  1.853X10^-2^ |
| OY56 | 767-1282  1118-1190 | OY175 (GU111974)  CLCuMB-[IN:DAB1:95]( AJ316037) | OYSOK3 (GU111992)  TbLCB-[PK:BAH:99]( AJ316034) | 2.4X10^-3^  NS | 6.611X10^-6^  NS | 4.611X10^-5^  1.105X10^-2^ | 3.690X10^-10^  3.453X10^-3^ | 2.953X10^-10^  NS | 1.372X10^-8^  1.853X10^-2^ |
| OYNUN | 759-1331  1110-1181 | OY175 (GU111974)  CLCuMB-[IN:DAB1:95]( AJ316037) | OYSOK3 (GU111992)  TbLCB-[PK:BAH:99]( AJ316034) | 2.4X10^-3^  NS | 6.611X10^-6^  NS | 4.611X10^-5^  1.105X10^-2^ | 3.690X10^-10^  3.453X10^-3^ | 2.953X10^-10^  NS | 1.372X10^-8^  1.853X10^-2^ |
| OYSOK3 | 624-792  1127-1198 | CroYVMB-Lucknow(EU604296)  CLCuMB-[IN:DAB1:95]( AJ316037) | OY168 (GU111990)  TbLCB-[PK:BAH:99]( AJ316034) | NS  NS | NS  NS | 2.846X10^-4^  1.105X10^-2^ | 3.641X10^-3^  3.453X10^-3^ | NS  NS | NS  1.853X10^-2^ |
| OY142 | 619-791  1110-1182 | CroYVMB-Lucknow(EU604296)  CLCuMB-[IN:DAB1:95]( AJ316037) | OY168 (GU111990)  TbLCB-[PK:BAH:99]( AJ316034) | NS  NS | NS  NS | 2.846X10^-4^  1.105X10^-2^ | 3.641X10^-3^  3.453X10^-3^ | NS  NS | NS  1.853X10^-2^ |

Footnote to Supplementary Table 1.

NS – no significant recombinant sequences detected.
